# Non Obstetric Causes and Presentation of Acute Abdomen among the Pregnant Women 

**Published:** 2014-09

**Authors:** Monoarul Haque, Farah Kamal, Shahanaz Chowdhury, Monir Uzzaman, Itrat Aziz

**Affiliations:** 1 Department of Community Nutrition, Bangladesh University of Health Sciences (BIHS), Dhaka, Bangladesh; 2 Department of Community Medicine, Noakhali Medical College, Noakhali, Bangladesh; 3 Department of Community Medicine, Bangladesh University of Health Science, Dhaka, Bangladesh; 4 Department of Gynecology and Obstetrics, Bangabandhu Sheikh Mujib Medical University (BSMMU), Dhaka, Bangladesh

**Keywords:** Acute Abdomen, Epigastric Pain, Aggravating and Relieving Factor

## Abstract

**Objective:** To identify the non-obstetric causes and presentation of acute abdomen among pregnant women.

**Materials and methods:** This was a cross sectional hospital-based study among 128 pregnant women by face to face interview using a semi-structured questionnaire. This study was conducted at the Gynecology & Obstetric Ward of 250 Bed General Hospital, Noakhali, Bangladesh, from January to August 2013. Data were analyzed by a software package used for statistical analysis (SPSS) version 11.5 (SPSS, Inc., Chicago, IL, USA).

**Results:** Mean age of participants was 25±4 years. Our findings showed that 81% were Muslim, 67% were lower middle income group, as well as 47% completed primary level of education. The results revealed that 28% had biliary ascariasis, 24% had peptic ulcer disease and 10% had lower urinary tract infection. We also found that 6% had acute pyelonephritis, 6% had acute gastroenteritis, 6% had acute cholecystitis, 6% had acute appendicitis, 2% had acute pancreatitis, 3% had choledocolithiasis, 2% had ovarian solid mass, 2% had twisted ovarian cyst, 4% had renal colic, and 1% had renal calculus. In non-obstetrical presentation of acute abdomen, the study found that 84% of respondents complained their pain lasting more than 24 hours. Besides, half of respondents felt pain in epigastrium and right hypochondrium. Cramping, prickling and aching type of pain were more, while 66% suffered from continuous pain. Our results also showed that 73% did not explain any aggravating factor and relieving factor, and the rest said food, fasting state and position change aggravated pain as well as relieved pain.

**Conclusion:** The study concludes that precise diagnosis of the acute abdomen in pregnant women by continual updating of abdominal assessment knowledge, and clinical skills is necessary in the management of abdominal pain in obstetric settings.

## Introduction

Abdominal pain is a common complaint of female inpatients and outpatients of all ages (1). Abdominal pain during pregnancy presents unique clinical challenges. First, the differential diagnosis during pregnancy is extensive, in that the abdominal pain may be caused by obstetric or gynecologic disorders related to pregnancy, as well as by intra abdominal diseases incidental to pregnancy. Second, the clinical presentation and natural history of many abdominal disorders are altered during pregnancy. Third, the diagnostic evaluation is altered and constrained by pregnancy. For example, radiologic tests and invasive examinations raise issues of fetal safety during pregnancy. Fourth, the interests of both the mother and the fetus must be considered in therapy during pregnancy (2). All these diagnostic uncertainties perpetuate the delay in decision making awaiting clear-cut symptoms and signs. Ironically, this delay when prolonged carries a high risk to the mother and the fetus (3). Abdominal pain in pregnancy poses a diagnostic and management challenge to the attending physician. Many causes are specific to pregnancy, but conditions affecting the non-pregnant woman can also complicate pregnancy. (4)Identifying the cause is influenced by the anatomical and physiological changes of pregnancy (4). Acute abdomen in pregnancy may be caused by various illnesses not related to pregnancy e.g. appendicitis, ileus, peduncular torsion of ovarian cyst, and acute cholecystitis and cholangitis. Acute pancreatitis and ureterolith, which also cause acute abdomen, are conservative treatment cases. The commonest cause of acute abdomen in pregnancy is acute appendicitis followed by acute cholecystitis (3). The character, severity, localization, or instigating factors of abdominal pain often vary with time. When the diagnosis and therapy is uncertain, close and vigilant monitoring by a surgical team, with frequent abdominal examination and regular laboratory tests, can often clarify the diagnosis. Occasionally, the pregnancy is not known by the patient or is not revealed to the physician, particularly in early pregnancy, when physical signs are absent. The physician should be vigilant for possible pregnancy in a fertile woman with abdominal pain, particularly in the setting of missed menses, because pregnancy is in the deferential diagnosis, clinical evaluation, and mode of therapy. Pregnancy tests should be performed early in the evaluation of abdominal pain in a fertile woman (2). When diagnosis and symptom control fail after 6-8 hours, a multidisciplinary approach should be considered. The safety and the possibility of a systematic cross-sectional evaluation of the entire abdomen have been considered as important reasons for the use of magnetic resonance imaging (MRI) in pregnancy with intractable pain. An appropriate laparoscopic surgery is now proving to be as safe as open surgery in pregnancy. Updating knowledge and assessment skills is essential in the management of abdominal pain in obstetric triage settings (4).

In day to day practice, physicians have to face pregnant women presenting acute abdomen. Maximum abdominal pain is not due to pregnancy itself. By identifying non obstetric causes of acute abdomen, it will be helpful for practitioners to think about this type of pain in pregnant women. In such condition presenting patterns of non obstetric causes of acute abdomen will be helpful in making differential diagnosis and making the plan of investigations to reach the final diagnosis. We aimed to identify the precise diagnosis of the acute abdomen in pregnant women.

## Materials and methods

A descriptive cross sectional hospital-based study was conducted among 128 pregnant women with complains of abdominal pain admitted to the Gynecology & Obstetric Ward of 250 Bed General Hospital, Noakhali, Bangladesh, from January to August 2013. Respondents selected purposively were willing to participate and to provide required information. We excluded pregnant women with true labor pain who were unwilling to give consent. An open ended semi structured questionnaire was used to solicit information on socio-demographic characteristics, non obstetrical causes of acute abdomen, non obstetrical presentation of acute abdomen and associated clinical features of acute abdomen. Data were checked thoroughly for consistency and completeness. All analysis was done by appropriate statistical methods using Statistical package for Social Sciences (SPSS) version 11.5 (SPSS, Inc., Chicago, IL, USA).

## Results

The cross-sectional study was conducted to assess the causes and the presentation of acute abdomen among pregnant women admitted in 250 Beds General Hospital, Noakhali, Bangladesh.

Table 1 shows that age range of respondents were 20-24 years in 41%, 25-29 years in 37%, 30-34 years in 21%, and 35-39 years in 1%. The minimum age groups of respondents were 20, and maximum age groups were 35 with mean age of 25.24 and standard deviation of 3.71. Majority of respondent (81%) were Muslim, 14% were Hindu, as well as 5% were Buddhist and Christian. Our results showed that 67% were lower middle income group (5361-21270), 14% were upper middle income group (21270-65761), and 19% were low income group according to 2006 Gross National Income (GNI) per capita and using the calculation of World Bank. It also shows that 47% completed primary education, 43% were illiterate, and only 2% completed higher secondary. It shows that 50%, 49% and 1% of respondents lived in nuclear, joint and third generation family, respectively. Regarding housing status, 61% of respondents lived tin shed house, 22% in semipacca house and 10% in paca house.

**Table 1:** Distribution of respondents according to socio-demographic characteristics (n=128)

	**Frequency**	**Percentage**
Age (Mean= 25.243.71)		
20-24	53	41
25-29	47	37
30-34	27	21
35-39	1	1
Religion		
Muslim	103	81
Hindu	18	14
Buddhist	3	2
Christian	4	3
Monthly family income		
<5360	24	19
5361-21270	86	67
21271-65761	18	14
>65761	0	0
Education		
Illiterate	55	43
Primary	60	47
Secondary	10	8
Higher-secondary	3	2
Family type		
Nuclear	64	50
Joint	63	49
Third generation	1	1
Housing status		
Kancha	9	7
Tinshed	78	61
Semipacca	28	22
Pacca	13	10
Total	128	100

Table 2 shows that 28%, 24%, 10% of respondents had biliary ascariasis, peptic ulcer disease, lower urinary tract infection, respectively. We also found that 6% had acute pyelonephritis, 6% had acute gastroenteritis, 6% had acute cholecystitis, 6% had acute appendicitis, 2% had acute pancreatitis, 3% had choledocolithiasis, 2% had ovarian solid mass, 2% had twisted ovarian cyst, 4% had renal colic, and 1% had renal calculus. 

Table 3 shows that 84% of respondents complained their pain lasting more than 24 hours. Besides, half of respondents felt pain in epigastria and right hypochondrium. Cramping, prickling and aching type of pain were more, while 66% of respondents suffered from continuous pain. 

**Table 2:** Distribution of the respondents according to non obstetrical causes of acute abdomen (n=128)

**Non obstetrical causes**	**Frequency**	**Percentage**
Biliary ascariasis	36	28
Peptic ulcer disease	31	24
Lower UTI	13	10
Acute pyelonephritis	8	6
Acute gastroenteritis	8	6
Acute cholecystitis	7	6
Acute appendicitis	7	6
Acute pancreatitis	3	2
Choledocolithiasis	4	3
Ovarian solid mass	2	2
Twisted ovarian cyst	3	2
Renal colic	5	4
Renal calculus	1	1
Total	128	100

**Table3:** Distribution of respondents according to non obstetrical presentation of acute abdomen (n=128)

**Non obstetrical presentation**	**Frequency**	**Percentage**
Pain duration		
<24 hours	20	16
>24 hours	108	84
Site of pain		
Epigastrium	36	28
Rt. Hypochondrium	46	36
Rt. Lumber	10	8
Lf. Lumber	4	3
Rt. Ileac fossa	9	7
Lf. Ileac fossa	3	2
Umbilical	8	6
Hypogastrium	12	9
Type of pain		
Cramping	45	35
Prickling	29	23
Stabbing	5	4
Throbbing	12	9
Aching	37	29
Character of pain		
Continuous	84	66
Periodic	44	34
Total	128	100

Table 4 shows that 73% of respondents did not explain aggravating factor and relieving factor, while the rest said food, fasting state and position change aggravated pain as well as relieved pain. Besides, 72%, 52%, 83%, 76% and 100% of respondents had no fever, vomiting, urinary frequency, constipation and vaginal bleeding, respectively.

**Table 4:** Distribution of respondents according to associated clinical features of acute abdomen (n=128)

**Associated presentation**	**Frequency**	**Percentage**
Aggravating factor		
Unexplained	94	73
Food	13	10
Fasting	14	11
Change of posture	7	6
Relieving factor		
Unexplained	94	73
Food	14	11
Fasting	13	10
Change of posture	7	6
Fever		
Yes	36	28
No	92	72
Vomiting		
Yes	61	48
No	67	52
Urinary frequency		
Yes	22	17
No	106	83
Constipation		
Yes	31	24
No	97	76
Vaginal bleeding		
Yes	0	0
No	128	100
Total	128	100

Table 5 reveals that anemia was present among 58% of respondents, but nobody had jaundice and very few had dehydration and diabetes. Besides, nobody had sexually transmitted diseases and HbsAg was negative among 98% of respondents.

## Discussion

Abdominal pain is a common complaint of female inpatients and outpatients of all ages, including women during their childbearing years, and thus often occurs during pregnancy. Abdominal pain during pregnancy presents unique clinical challenges. First, the differential diagnosis during pregnancy is extensive, in that the abdominal pain may be caused by obstetric or gynecologic disorders related to pregnancy, as well as by intraabdominal diseases incidental to pregnancy (1). The present study shows that 28%, 24%, 10% of respondents had biliary ascariasis, peptic ulcer disease, lower urinary tract infection. Besides, acute pyelonephritis, acute gastroenteritis, acute cholecystitis, acute appendicitis, acute pancreatitis, choledocolithiasis, ovarian solid mass, twisted ovarian cyst, twisted ovarian cyst, renal colic and renal calculus were in 6%, 6%, 6%, 6%, 2%, 3%, 2%, 2%, 4% and 1% of respondents, respectively .

**Table 5:** Distribution of respondents according to clinical examination and investigations (n=128)

**Clinical examination**	**Frequency**	**Percentage**
Anemia		
Present	54	42
Absent	74	58
Jaundice		
Present	0	0
Absent	128	100
Dehydration		
Present	6	5
Absent	122	95
Diabetes		
Present	5	3
Absent	123	97
VDRL		
Reactive	0	0
Nonreactive	128	100
HbsAg		
Positive	3	2
Negative	125	98
Total	128	100

However, another study showed that in a series of 48,482 pregnancies, laparotomy was undertaken 74 times for conditions not associated with pregnancy (1 in 655 pregnancies). It showed no abnormality in 26 cases; ovarian cysts and acute appendicitis were the commonest pathological findings. The preoperative diagnosis was proved correct in 53% of cases, and in 66.2% laparotomy proved to be necessary for an alternative diagnosis. The fetal loss rate after surgery was 23%. Spontaneous abortion was more likely in the presence of peritonitis, with fluid in the peritoneal cavity, or when operative procedures involving the ovary were performed within the first trimester. The risk of precipitating labour following diagnostic laparotomy is negligible, provided no unnecessary surgical maneuvers are undertaken (5). A study was done among Saudia Arabia population to calculate the frequency of acute abdomen in pregnancy due to non-obstetric causes, to discuss the etiology of the high incidence, to discuss how pregnancy altered the symptomatology of acute abdomen and to evaluate the result of early surgical intervention and use of tocolytics on maternal and fetal health and they showed that the frequency of acute abdomen in pregnancy due to non-obstetric causes in this population is 0.39% which is high in comparison to other studies and the etiology is multifactorial. Resemblance of early acute abdomen symptoms like nausea, vomiting to those of normal pregnancy and the anatomical displacement of abdominal organs by the pregnant uterus greatly masked the clinical picture and enhanced surgical delay awaiting definitive criteria for surgical intervention. This delay significantly increased maternal morbidity (p < 0.05) and resulted in a poor fetal outcome. Those who had early surgical intervention had a better perinatal outcome (p< 0.001) and decreased maternal morbidity (p< 0.05). Although tocolytics were used, they proved to be ineffective, altered the maternal clinical picture and had fetal side-effects (3).

Section of Hepatology, University of Colorado, School of Medicine, Denver, stated that the gallbladder and gut should be viewed as hormonally responsive organs the normal physiology of which may be altered by the hormones of pregnancy. The gallbladder enlarges and empties sluggishly in response to meals during pregnancy. Small bowel transit is slowed, and the resting pressure of the lower esophageal sphincter is reduced. All these effects are reversed by delivery; motility reverts toward normal in the postpartum period. The rapid return of normal motility suggests that the effects of pregnancy are hormonally related. Most studies have demonstrated that progesterone, not estrogen, may be the hormone responsible. Although incompletely defined, one mechanism of the effects of pregnancy on motility may be progesterone-induced inhibition of the mobilization of intracellular calcium within smooth muscle cells (6). My study also found that 73% of respondents did not explain aggravating factor and relieving factor, and rest of the respondents said that food, fasting state and position change aggravated pain as well as relieved pain. Besides, 72%, 52%, 83%, 76% and 100% respondents had no fever, vomiting, urinary frequency, constipation and vaginal bleeding, respectively. Ovarian torsion (OT) is a well-known yet poorly recognized clinical entity that can involve the tube, ovary, and ancillary structures either separately or together (7). It is the fifth most common gynecologic emergency, with a reported incidence of 3% in one series of acute gynecologic complaints (7-8). However, the diagnosis of OT can be difficult to make. The majority of women with OT are seen in the emergency department with an acute onset of abdominal pain (8-11). A large subset of these patients also experienced the associated nausea or vomiting (11-12). The differential diagnosis for OT is broad and includes many other emergency causes for abdominal pain, such as ectopic pregnancy, pelvic inflammatory disease, appendicitis, diverticulitis, ovarian cyst, and renal colic (7,12). Early diagnosis and laparoscopic treatment is recommended for suspected OT, particularly to salvage the ovary and adnexa in women desiring to maintain fertility. Yet, an ovarian salvage rate of less than 10% has been reported (7,10)**.** Although ovarian necrosis could potentially be fatal, no deaths resulting from a missed diagnosis have been reported. However, if nonspecific severe pain is seen, OT can be an important differential consideration in the evaluation of a potentially surgical abdomen. A fifteen year review on ovarian torsion stated that pain characteristics were variable: the onset was sudden in 51 (59%); “sharp” or stabbing in 61 (70%); and radiated to the flank, back, or groin in 44 (51%) patients. Only 3 had peritoneal signs at presentation. The majority of patients (70%) had nausea or vomiting. Fever was rare (2 patients). OT was considered in the admitting differential diagnosis in 41 (47%) patients. An enlarged ovary (>5 cm) was found in 77 (89%) patients at surgery. Only 26 patients had surgery within 24 hours. In 8 (9%) patients, detorsion was possible; of these, 3 had surgery within 24 hours (13). On the other hand, my study found that 84% of respondents complained their pain lasting more than 24 hours. Besides, half of respondents felt pain in epigastrium and right hypochondrium. Cramping, prickling and aching type of pain were more, while 66% of respondents suffered from continuous pain. 

Biliary ascariasis was reported in the literature as early as 1946 or earlier (14). The first report of biliary ascariasis in North America or United Kingdom was published in 1977 (15). Since then many report of biliary ascariasis published worldwide, especially in Asia and Latin America (16). Another study showed that biliary ascariasis is one of the common causes of acute abdominal pain among the hospitalized female patients in their clinical experience, The most common symptoms are acute upper abdominal pain, nausea, vomiting, occasionally fever and jaundice simulating acute cholecystitis. Common complications of biliary ascariasis are acute cholecystitis, acute cholangitis due to accompanying bacterial contamination, acute pancreatitis and liver abscess. (17) Hepatobiliary lithiasis can occasionally be seen as a remote complication (18). Another review stated that ascariasis, a helminthic infection of humans, is the most common parasitic infestation of the gastrointestinal tract. It infects about 25% of the world's population; around 20 thousand deaths occur per year from an adverse clinical course of the disease. This review is focused on biliary ascariasis, examining in some detail the pathogenesis of the disease with special reference to postcholecystectomy ascariasis and related issues. Although an endemic disease of tropical and subtropical countries, increasing population migration facilitated by fast improving communication facilities demands that clinicians everywhere be familiar with the clinical profile and management of biliary ascariasis (14).

## Conclusion

This study has yielded some valuable information. On the basis of the findings of this study, it is clear that the precise diagnosis of the acute abdomen in pregnant women is necessary. Differential diagnosis should be carefully done to take appropriate measures. As so, it demands continual updating of abdominal assessment knowledge and clinical skills in the management of abdominal pain in obstetric settings.
